# Detecting Medication-Taking Gestures Using Machine Learning and Accelerometer Data Collected via Smartwatch Technology: Instrument Validation Study

**DOI:** 10.2196/42714

**Published:** 2023-05-04

**Authors:** Chrisogonas Odero Odhiambo, Lukacs Ablonczy, Pamela J Wright, Cynthia F Corbett, Sydney Reichardt, Homayoun Valafar

**Affiliations:** 1 Department of Computer Science and Engineering University of South Carolina Columbia, SC United States; 2 Honors College University of South Carolina Columbia, SC United States; 3 Advancing Chronic Care Outcomes through Research and iNnovation Center, College of Nursing University of South Carolina Columbia, SC United States

**Keywords:** machine learning, neural networks, automated pattern recognition, medication adherence, ecological momentary assessment, digital signal processing, digital biomarkers

## Abstract

**Background:**

Medication adherence is a global public health challenge, as only approximately 50% of people adhere to their medication regimens. Medication reminders have shown promising results in terms of promoting medication adherence. However, practical mechanisms to determine whether a medication has been taken or not, once people are reminded, remain elusive. Emerging smartwatch technology may more objectively, unobtrusively, and automatically detect medication taking than currently available methods.

**Objective:**

This study aimed to examine the feasibility of detecting natural medication-taking gestures using smartwatches.

**Methods:**

A convenience sample (N=28) was recruited using the snowball sampling method*.* During data collection, each participant recorded at least 5 protocol-guided (scripted) medication-taking events and at least 10 natural instances of medication-taking events per day for 5 days. Using a smartwatch, the accelerometer data were recorded for each session at a sampling rate of 25 Hz. The raw recordings were scrutinized by a team member to validate the accuracy of the self-reports. The validated data were used to train an artificial neural network (ANN) to detect a medication-taking event. The training and testing data included previously recorded accelerometer data from smoking, eating, and jogging activities in addition to the medication-taking data recorded in this study. The accuracy of the model to identify medication taking was evaluated by comparing the ANN’s output with the actual output.

**Results:**

Most (n=20, 71%) of the 28 study participants were college students and aged 20 to 56 years. Most individuals were Asian (n=12, 43%) or White (n=12, 43%), single (n=24, 86%), and right-hand dominant (n=23, 82%). In total, 2800 medication-taking gestures (n=1400, 50% natural plus n=1400, 50% scripted gestures) were used to train the network. During the testing session, 560 natural medication-taking events that were not previously presented to the ANN were used to assess the network. The accuracy, precision, and recall were calculated to confirm the performance of the network. The trained ANN exhibited an average true-positive and true-negative performance of 96.5% and 94.5%, respectively. The network exhibited <5% error in the incorrect classification of medication-taking gestures.

**Conclusions:**

Smartwatch technology may provide an accurate, nonintrusive means of monitoring complex human behaviors such as natural medication-taking gestures. Future research is warranted to evaluate the efficacy of using modern sensing devices and machine learning algorithms to monitor medication-taking behavior and improve medication adherence.

## Introduction

### Background

Over 3 decades of international research has indicated that complete models of human health comprise complex interactions of biological, behavioral, and environmental factors. While substantial technological advances exist in the study of the biological and environmental bases of diseases, there have been relatively minor advances in technologies for characterizing human behaviors that influence health. Technological devices have pervaded and revolutionized much of our social and private lives, yet their implementation and use in health care remain sparse. In particular, the innovative use of existing, widely used, and commercially available technologies to influence health-promoting behaviors has been underexplored. Adapting smart technologies, such as phones and watches, has the potential to initiate more effective health-promoting interventions for behaviors such as weight loss, physical activity, and medication adherence. Adapting these devices to promote healthier behavior requires solving the crucial problem of characterizing and monitoring human behavior in a way that will be useful, unobtrusive, and personally relevant. Once resolved, the subsequent steps in developing optimal and personalized interventions can be explored.

Better understanding of behavioral activities such as eating, smoking, sleeping, exercising, and medication taking can have a substantial impact on population and individual health, with the potential to significantly reduce overall health care costs worldwide. In this study, we focused on the global public health challenge of medication adherence, as only approximately 50% of people adhere to their medication regimens [1]. Medication adherence, defined as taking medicines according to decisions agreed upon between prescribing health care professionals and patients [2,3], is a complex human behavior critical for the management of chronic health conditions. Studies have identified forgetfulness as the main reason for nonadherence to many long-term medicines [4]. To address forgetfulness, findings from a meta-analysis of medication adherence interventions among adults demonstrated that linking medication taking with existing daily routines and using behavioral strategies (eg, prompts to take medication) are the most effective approaches to promote adherence [4]. Smartphone apps and other technology-based reminders have also shown promising results in promoting medication adherence [5-8]. However, practical mechanisms to determine whether a medication has been taken or not, once people are reminded, remain elusive.

Different methods, both direct and indirect, exist to measure whether a medication has been taken. However, none are considered a gold standard. Direct measurements, such as clinical biomarker specimens or metabolites from pharmaceutical metabolism and direct observations of medication taking, can be expensive and impractical, especially in large population settings [9]. Indirect methods, such as pill counts, electronic monitoring, and self-reporting, offer simpler alternatives but, at best, approximate adherence through proxy data that can be initially overestimated with even less reliability over time [10]. An ideal method to measure medication adherence should be accurate, affordable, and practical (ie, easy to implement).

Recent advances in sensor technology and artificial intelligence (AI) present an innovative opportunity to measure medication adherence objectively, unobtrusively, and conveniently. Wearable devices such as smartwatches may offer the platform to observe medication adherence [11-13]. From a modest 37 million units in 2016, smartwatch shipments worldwide are projected to increase by 253 million units by 2025 [14]. Smartwatches are likely to become as pervasive as cell phones, as their prices continue to decline and they become more advanced with additional sensors and mobile health applications. Anticipating this trend, our team investigated smartwatch use not only for medication reminders but also as a strategy for monitoring medication adherence. In this report, we present an artificial neural network (ANN) approach [15] that can detect the complex behavior of medication taking, called the natural medication-taking event (nMTE), with as high as 95% accuracy using sensor data available from common smartwatches.

### Previous Work

The use of sensors to automatically detect human activities was pioneered by the work of the Neural Network house and was reported in the late 1990s [16-19]. Several studies have illustrated how smartwatches can be used to monitor and detect human motions of interest, such as smoking, [20-23] or falls among older adults [24-27]. Independent reports [13,28-32] have also confirmed the usability of smartwatches to detect other human motion behaviors, such as eating, physical activity, and foot movement. In the last decade, inspired by the introduction of smart wearable devices, human activity recognition has expanded to include activities such as cigarette smoking [20,22], falls [33-35], and sleep [36,37]. Sleep activity has been studied further using sensor data obtained from electroencephalograms and electromyogram devices to develop neural network models [38]. To detect medication taking, numerous approaches and technologies have been introduced, including experimental devices worn on wrists [15,39-41], sensors worn around the neck to detect swallowing [42-44], and vision modules embedded in smart environments such as Microsoft’s EasyLiving project. [45,46]^,^ The EasyLiving project showcased the early investigations into context-aware computing using an array of video-capture devices instead of more traditional physical sensors. By using several vision modules in each room, the system could identify motions, people, gestures, and the surrounding environment. The project also focused on the geometric relationships between people, places, and things to build context and form interaction information that would associate objects with their likely use, which could later be used in a more intelligent system for behavior prediction. Although vision-based medication adherence monitoring is a viable human activity recognition method, users’ privacy concerns and the identifiable nature of the data were strong deterrents to the adoption of this method. In contrast, sensor-based smartwatches provide a scalable and practical platform for convenient, unobtrusive, and secure study of human behavior in natural settings (eg, people’s homes). Our previous work highlighted the potential of smartwatches to monitor medication-taking events (MTEs) under protocol-guided (scripted) conditions (scripted MTEs [sMTEs]), where all participants followed the same method of taking their medication (eg, use of the right hand to perform most activities) [15]; however, an nMTE may significantly depart from the scripted method. For example, a person may prefer to take the pill with their right hand while drinking water with their left hand. To establish a more practical application of this technology, we explored the feasibility of detecting unscripted events (nMTEs), which extends our previous study [15]. The nMTE may be more generalizable, and thus, it is a more powerful approach to accurately monitor medication taking and measure medication adherence. The nMTE model has the potential to be an effective intervention tool that can increase adherence, reduce accidental instances of overmedication, and be used for medication adherence monitoring by support persons or health professionals.

### Objective

The purpose of our study was to test the capabilities of our detection model; we used sensor data from MTEs (sMTEs and nMTEs), other similar activities (eg, eating and smoking), and a dissimilar activity of jogging.

## Methods

### Participant Recruitment and Data Collection Process

#### Overview

The study was conducted during the height of the COVID-19 pandemic and required substantial departure from the traditional means of engaging human participants in sensor recognition studies, which have primarily occurred in laboratory settings. The participants (N=28) were recruited using snowball sampling. The inclusion criteria were adults willing and able to complete the study protocol following training. The exclusion criteria were any type of movement disorder (eg, Parkinson disease) or paresis (eg, muscular weakness owing to conditions such as stroke). An appointment was made with each potential participant to explain the purpose, benefits, and risks of the study and to address any questions or concerns. After obtaining informed consent, the participants completed a demographic questionnaire and then received a packet with a smartwatch and phone, 2 charger cables, a user manual, a pill bottle, and placebo medication. The user manual was presented and discussed in detail to the participants. Before data collection, the research team members had an internet-based meeting with each participant to train them to collect and transfer the data, which culminated in participants properly demonstrating the activities.

#### Collected Data

**Figure 1:**
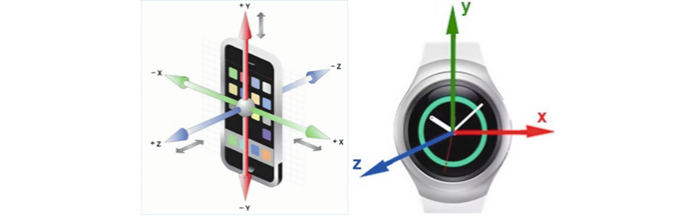
An illustration of smartphone and smartwatch accelerometer axes.

Figure 1 depicts the smartphone and smartwatch with triaxial sketches. The smartwatch was used to collect data, and the phone was used to upload the data to cloud storage. The participants wore the watch on their right wrist for sMTEs and on their wrist of preference for nMTEs. The collected data contained hand-motion accelerometer sensor logs of the triaxial values recorded by the watch at a sampling rate of 25 Hz. The data included the time stamp, orientation, and acceleration of the hand during medication-taking activities. The xyz-sensor values were logged into a CSV file by the medication-taking app on the watch. The file was periodically and asynchronously moved to the phone via Bluetooth.

#### Data Collection Protocol

When the participants received their study supply packet, we collected demographic data and scheduled a protocol training session. Following the medication-taking training sessions, the participants independently completed the data collection in their homes. The exercise comprised the first week (ie, 5 days) of performing medication-taking behaviors using the participant’s natural way of taking medications (nMTEs) and the second week of performing medication-taking behaviors according to a scripted protocol (sMTEs) [15]. Each participant was directed to record 10 nMTE gestures per day for the first 5 days and then 10 sMTE gestures for the next 5 days. In total, 1400 nMTEs and 1400 sMTEs were collected, tallying 2800 gestures.

To enable seamless transfer of data from the watches to cloud storage, each watch was paired with a smartphone. A custom Android application called MedSensor, a software developed by the research team, was installed on both the watch and phone. At the participants’ convenience, the watch data were transferred to the phone via Bluetooth connectivity.

#### Closure

After collecting and transferring 10 days of collected data, the participants returned the smartwatch and phone to the study project coordinator and received a US $25 gift card as an incentive. The smart devices were sanitized according to the Centers for Disease Control and Prevention (CDC) guidelines before use by the other participants.

### Ethical Considerations

The study was reviewed by the University of South Carolina Institutional Review Board and received exemption from Human Research Subject Regulations (Pro00101203). Potential participants were informed of the study purposes, potential risks and benefits, and their rights as research participants, including the voluntary nature of participation. All participants provided verbal consent before study participation.

### Data Preparation and Annotation

#### Overview

The proper use of data in supervised machine learning (ML) approaches requires reliable annotation of the data. The process required a *supervisor* (an expert) to identify and define the gestures of interest to be used during the training of ML models. To develop an understanding of which signal constitutes an MTE, in our previous work, we used the sMTE data collected to understand the individual components of the medication-taking gesture. The exact details of the scripted gesture can be found in our previous report [15]. Using this information, the team members identified and annotated the individual gestures of the nMTEs. The process of gesture identification and annotation was accelerated by having participants self-report MTEs on their smartwatches that included time stamps indicating the beginning and ending of each MTE.

The raw data files logged at the cloud repository consisted of a time stamp that included hours, minutes, seconds, and milliseconds; a date that included day, month, and year; and the x, y, and z components of the accelerometer data. The second file contained the time stamps corresponding to the start and end of each MTE reported by the participant. In theory, the self-reported MTE should be sufficient to identify the gesture of interest (ie, medication taking). However, in practice, participants may erroneously report the activity, or the time stamps may only approximate the correct start and end times of the activity. Therefore, each gesture is visually confirmed to ensure high-quality data. A separate utility program was developed to facilitate this process and to create the final usable data [47]. After this final step, the data files were presented in a usable format for training and testing the AI model.

#### Secondary Data Acquisition and Preparation

This study integrated accelerometer data from 4 different human activities (Table 1), with the primary focus on recognizing nMTEs as recorded by smartwatches (Polar M600, Asus Zenwatch, Motorola, and TicWatch), as described in the previous section. The sMTE data were recorded by each participant wearing the smartwatch on the right wrist, then sequentially performing the mini-activities of medication taking (opening the bottle, dispensing pills to the right palm, placing pills in the mouth with the right hand, drinking water with the left hand, and closing the bottle). For the nMTE, the participant performed the same mini-activities in any sequence that is natural to them (ie, how they would typically take medicine). Smartwatch data for other behaviors (ie, smoking and eating) consisted of data reported in previous studies [48,49], whereas the MTE data were collected in this study using the protocol described in the previous section. The jogging data set, by contrast, is open public data from the Wireless Sensor Data Mining Laboratory [50] that were recorded using a smartphone strapped to the waist of the participant.

**Table 1:** Summary of all the data sets used in the study.

Activity	Data points, n	Gestures, n	Participants, n
Medication	824,000	2800	28
Eating	272,822	5434	6
Smoking	62,823	1279	12
Jogging	287,461	5883	27

#### Data Preprocessing and Standardization

Before integrating data from multiple studies, several normalization and standardization steps were performed. Specifically, attention was paid to the consistent standardization of the accelerometer data and sampling rate of the data. Because the devices used for data collection across all studies were Android devices (vs Apple devices), the sensor data followed a common frame of the x-, y-, and z-axes. As the next step, all data sets were processed to adhere to a sampling rate of 25 Hz by excluding data points (in the case of oversampling) or resampling based on the interpolation of the data (in the case of undersampling). To normalize for the different numbers of gestures per activity, the individual gestures were represented multiple times in our data set to provide a balanced representation of activities.

### Development of the ANN

#### Neural Network Platform and Architecture

**Figure 2:**
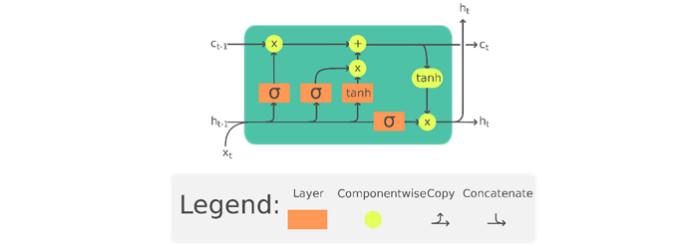
The long short-term memory cell can process data sequentially and keep its hidden state through time (reproduced from Chevalier [54], which is published under Creative Commons Attribution 4.0 International License [55]). c: memory cell; h: hidden state or output vector of the LSTM unit; x: input vector to the LSTM unit; tanh: activation function; subscript t indexes the time step.

The human activities of interest in this study—medication taking, eating (pizza), smoking, and jogging—each included a sequence of mini-activities that have temporal properties. The temporal property is a crucial gesture sequence component in the complex activity recognition. For example, the MTE comprised a series of mini-activities, namely (1) open-bottle and dispense-medicine; (2) hand-to-mouth, pill-into-mouth, and hand-off-mouth; and (3) pick-up-water, drink-water, lower-cup-to-table, and close-bottle. The temporal property (time stamp in this case) determines the sequence of the mini-activities, thereby determining the uniqueness of each complex activity. Considered through the lenses of a linguist, where the mini-activities form the words and the time stamp is the order of activities, the meaning or semantics of the full activity was determined by the syntax of this activity sentence [51]. On the basis of the architecture that incorporates long-term memory, the long short-term memory (LSTM) neural network is the best candidate for the implementation of human activity recognition systems. An LSTM neural network is an artificial recurrent neural network (RNN) architecture with feedback connections that facilitate awareness of past activities at the present time of the activity [52,53]. Figure 2 illustrates a typical LSTM cell where *x_t_* is the input vector to the LSTM unit; *h_t_* is the hidden state vector (or LSTM unit output vector); *c_t_* is the cell state vector; and *c_t−1_* is the cell input activation vector. In this study, our model contained 2 fully connected and 2 LSTM layers (stacked on each other), with 64 units each. The learning rate was set at 0.0025.

#### Training and Testing Procedure

The LSTM network was trained for 150 epochs using the annotated data while keeping track of accuracy and error. Network training is a process in which a ML algorithm is fed sufficient training data to learn from. The training requires multiple passes on the training data. Epoch refers to the total number of iterations of all the training data in one cycle for training the ML model. The batch size was maintained at 1024; batch size refers to the number of input samples that are passed to the network at once. The train and test data sets were partitioned in an 80:20 ratio, respectively, after the balancing procedure. We applied L2 regularization (Ridge Regression) to the model. The L2 penalty/force removes a small percentage of weights at each iteration, ensuring that weights never become zero. Consequently, the penalty reduces the chance of model overfitting.

Each recording data session may contain hours or days of sensor data. The immediate question to answer is what portion of this recording should be presented to the neural network to identify an activity. The LSTM network expects the training data input of fixed length, also referred to as the “window size.” In this study, a window size of 150 points (ie, 150 rows of sensor data logs) was empirically determined to provide an acceptable performance. At a sampling frequency of 25 Hz, this translates to approximately 1.5 seconds of contiguous recorded data. While the window size represents the portion of the raw data that should be in direct view of the ANN at the time of classification, any relevant past contextual information is saved in the internal cell of the LSTM architecture. The temporal exposure of the LSTM-RNN can be accomplished in various ways, including a sliding window of appropriate size. In this study, the sliding window size of 150 points was selected as the optimal compromise between the performance, simplicity, and responsiveness. The sliding window with overlap significantly transforms and reduces the training data set. Furthermore, the transformation assigns the most common activity (ie, mode) in the exposed window of 150 points as a label for the sequence. This is necessary because some windows may contain ≥2 activities, but the mode is considered to be the dominant or overriding activity. Consequential to the input definition, the data were reshaped into sequences of 150 rows, each containing x, y, and z values with 10 points of overlap between 2 consecutive windows. The desired output of the system was based on one-hot encoding of the labels to transform them into numeric values that can be processed by the model [56,57].

#### Evaluation of the Trained Network

During the training phase of an ANN, a single metric of performance needs to be defined to assess the network performance. The network performance metric is used by the operator to direct the network and improve the overall performance. In this study, we evaluated the performance of the classifiers using the accuracy metric defined in the equation 1. In equation 1, true-positive (TP) results represent the correctly classified positive examples; true-negative (TN) results represent the correctly classified negative examples; false-positive (FP) results represent negatives misclassified as positives; and false-negative (FN) results represent positives misclassified as false.

Accuracy = (TP + TN) / (TP + TN + FP + FN) **(1)**

As a network performance measure, the accuracy does not account for the bias arising from unbalanced data sets. To remove the effect of unbalanced data (unequal representation of different activities), data within each activity were repeated to arrive at an approximately equal number of representations for medication taking, eating, smoking, and jogging. Despite the adjustments to enforce data set balance, some data sets remained larger than others, potentially translating into a biased favor for the majority classes. Therefore, the study team considered the following additional evaluation criteria: precision, recall, F-measure, and specificity. Precision indicates the fraction of positive predictions that are truly positive. Recall (positive) indicates the fraction of all positive samples that are correctly predicted as positive by the classifier (TP rate). Recall (negative) indicates the fraction of all negative samples that are correctly predicted as negative by the classifier (TN rate).

Below are the formulas to compute the metrics:

Precision = TP / (TP + FP) **(2)**

Recall = TP / (TP + FN) **(3)**

F-measure is the combination of precision and recall. This is calculated as follows:

F-measure = ([1+ β^2^] × recall × precision) / (β^2^ recall + precision) **(4)**

where β is a weighting factor and a positive real number. It is used to control the importance of recall and precision.

Specificity = TN / (TN + FP) **(5)**

## Results

### Sample Demographics

Recruited participants (N=28) had a mean age of 27.25 (range 20-56) years, and 57% (16/28) were male. The majority were college students (21/28, 71.4%), single (24/28, 86%), and working at least part time (17/28, 61%). The sample represented racial diversity with 43% (12/28) Asian and 43% (12/28) White individuals, 10% (3/28) belonging to ≥2 races, and 4% (1/28) African American individuals [58]. Most participants (23/28, 82%) were right-hand dominant, whereas only 1 (4%) participant was ambidextrous.

### Visualization of Medication-Taking Protocol Gesture

**Figure 3:**
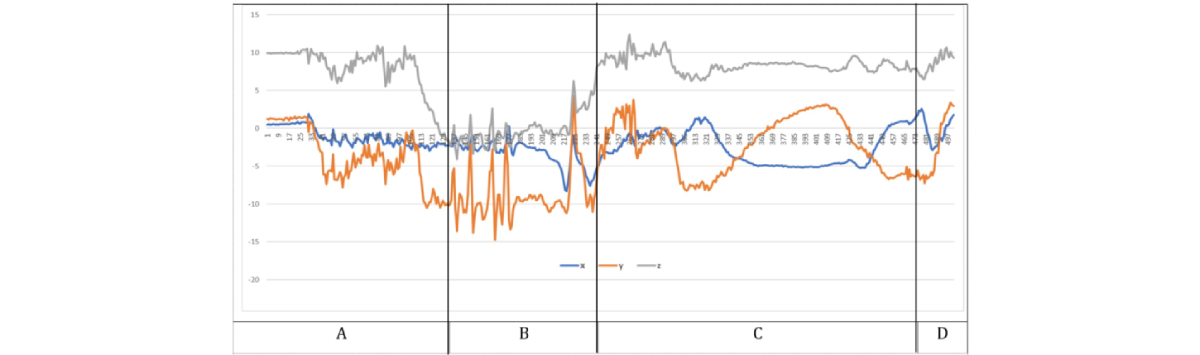
Atomic segmentations of the complex activity, the medication-taking event, corresponding to (A) open-bottle and dispense-medicine, (B) hand-to-mouth, pill-into-mouth and hand-off-mouth, (C) pick-up-water, drink-water, lower-cup-to-table and close-bottle, and (D) after the medication.

As the first step in performing activity recognition with wearable devices, a more detailed understanding of the gesture of interest needed to be developed. Figure 3 represents an example of an entire sMTEs recorded from a right-hand dominant participant. After careful and repeated examination of the gesture, sequential segments of the gesture were identified (Figure 3). When considering the portion of the gesture corresponding to water drinking (phase C), the gradual increase in the accelerometer’s y-axis depicts the beginning of the drinking phase. It can be used both as a hallmark of an MTE and for quantifying drinking duration.

**Figure 4:**
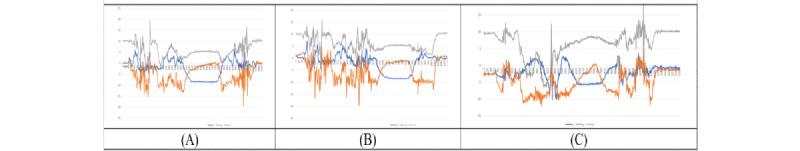
Illustration of medicine-taking protocol differences between (A) scripted gesture from user1, (B) natural gesture from user1, and (C) natural gesture from user2. The 3 recorded sessions illustrate the diversity in natural gestures and potential departure from the scripted gestures.

Our medication-taking study consisted of sMTEs and nMTEs. We based the scripted protocol on the natural behavior observed in most of our preliminary studies. Nevertheless, people’s nMTEs varied from the sMTEs as illustrated in Figures 4A, 4B, and 4C.

**Figure 5:**
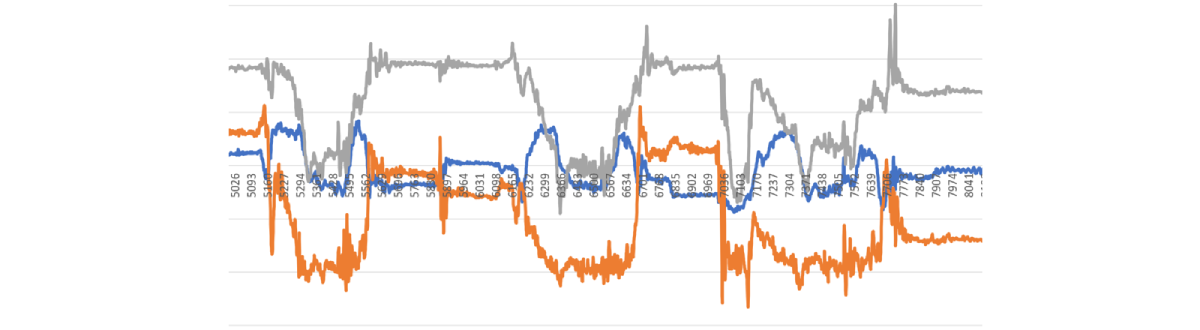
Three bites of pizza-eating activity.

**Figure 6:**
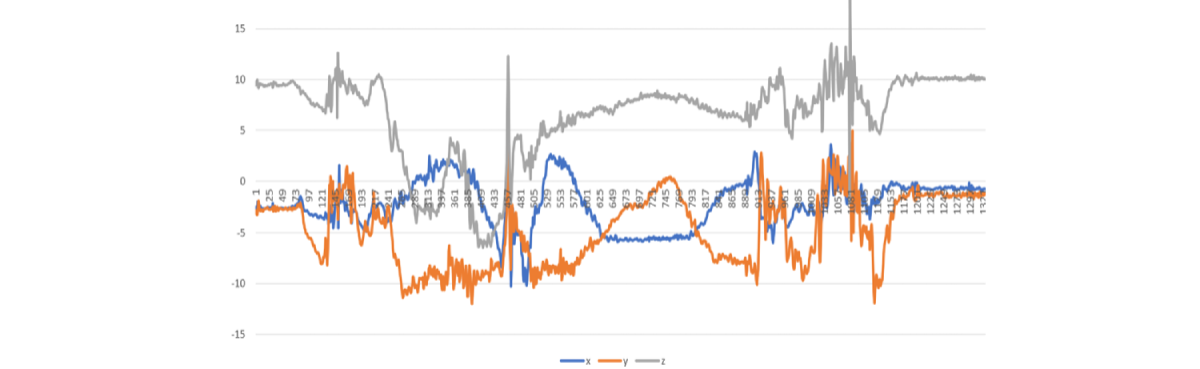
Single medication-taking event.

**Figure 7:**
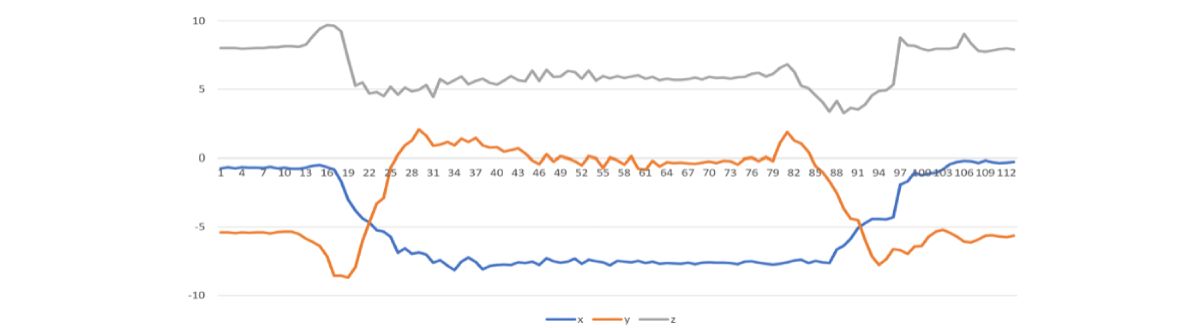
Single puff of smoking gesture.

**Figure 8:**
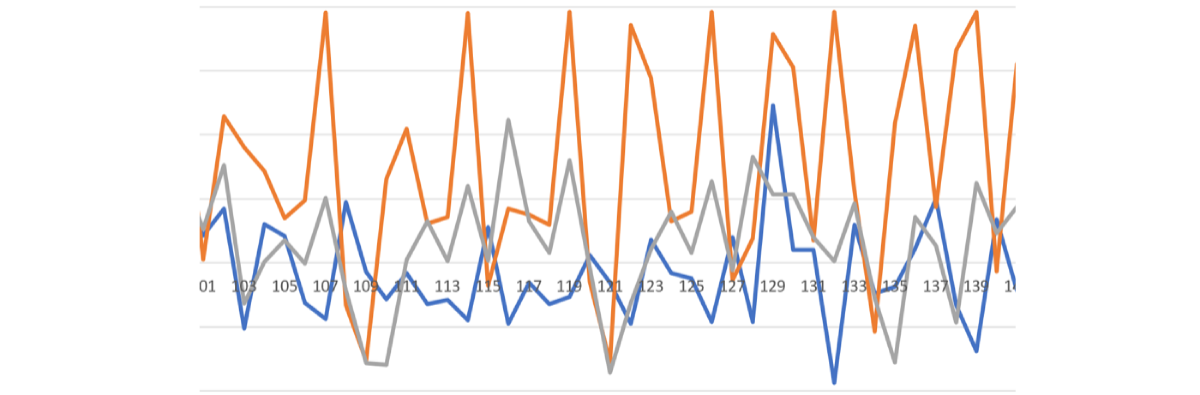
Jogging exercise gestures.

The visualizations in Figures 5-8 represent the unique signatures of the following activities: pizza eating, medication taking, smoking, and jogging activities.

It is important to highlight the challenging task of identifying nMTEs, given the gesture diversity in mini-activities among different participants. For instance, the simple method of drinking water between 2 participants can vary significantly as illustrated in Figures 4B and 4C. The participant in the Figure 4B performed the task of drinking water with a sudden removal of glass from the mouth, and the participant in Figure 4C removed the glass from their mouth more gradually. These differences in the individual mini-activities are the root of the challenges associated with smartwatch gesture detection. Nevertheless, the combined activities of medication taking are distinctive from the other activities as illustrated in Figures 5-8.

### Validation and Annotation of MTEs

The study of human behavior using wearable devices has several advantages over the traditional self-report methods. Specific to medication taking, self-reported adherence is known to be overestimated [59]. In comparison to self-reports, wearable devices can provide additional useful information such as the time of the day the medication was administered and the number of times the medication was taken in a day without incurring additional time, effort, or cost to the user. The natural gestures duration ranged between 5 and 331 seconds, with a mean of 18.47 (SD 14.34; median 17) seconds per gesture. The scripted gestures duration ranged between 4 and 686 seconds, with a mean of 20.11 (SD 20.65; median 18) seconds per gesture. Considering the average time needed to complete an MTE, outliers can be examined. We considered gestures longer than 100 seconds or shorter than 8 seconds as outliers. There were 6 scripted gestures and 2 natural gestures of a duration of ≥100 seconds. There were also 63 scripted gestures and 40 natural gestures of a duration of ≤8 seconds. In this study, we considered outliers (both natural and scripted) as gestures with a duration of ≤8 seconds for the lower category or ≥100 seconds for the upper category. To determine the outliers, we considered the mean and SD for natural gestures at 18.47 (SD 14.34) seconds and scripted gestures at 20.11 (SD 20.65) seconds. Fewer outliers were observed in the sMTEs than in nMTEs. For the lower category, a random sample of 20 out of 103 gestures was examined, and all were invalid gestures, indicating that the users probably annotated the start/stop gestures in quick succession. By contrast, for outliers of ≥100 seconds in duration, most contained ≥1 MTE gestures in most cases. To better understand the cause of these temporal discrepancies and therefore, validate or invalidate the reported gestures, each recording session was examined by a team member for validity. The results are presented in Table 2.

**Table 2:** Analysis of upper-category outliers in seconds.

Participants	Duration (seconds)	Observations	Correction
1	371	The participant reported 7 consecutively taken medications as 1 medication event.	Individual MTP^a^ events were separated by an ML^b^ supervisor.
1, 2, 3, 4, 5, and 6	162	One MTP event was observed with some unrelated activities at the beginning or end of the recording.	The unrelated portions of the recording were trimmed.
3	279	Comprises random activities that do not match medication-taking gesture pattern	—^c^

^a^MTP: medication-taking protocol.

^b^ML: machine learning.

^c^Significant aspect of human gestures (that in simulation of human gestures, participants are still bound to perform other random activities, probably out of interruption or disruption). This emphasizes the natural medication exercises where every activity happens in the context of other activities.

### ANN Training and Testing

**Figure 9:**
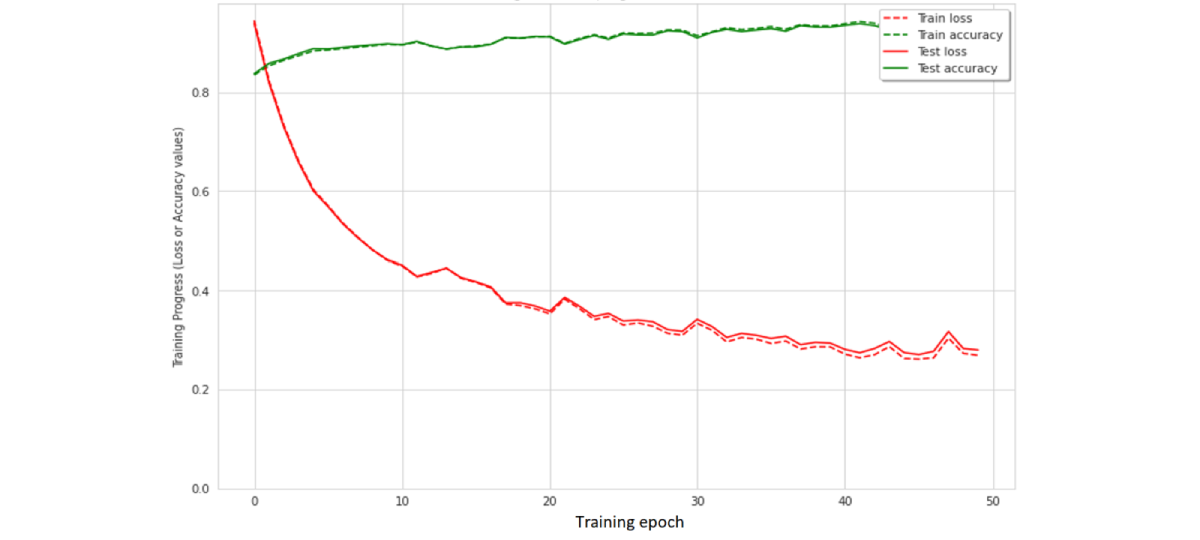
Training plot for the window size of 150 units. The configuration resulted in the best performance among the different models.

As an initial step in the training of a neural network, examining the learning curve can be instrumental. Figure 9 illustrates the learning curve of the designed LSTM-RNN after the proper treatment of the outlier data. The figure shows the training accuracy (depicted in green) for the training and testing sets (dashed and solid lines, respectively). Here, the consistently increasing values of accuracy is an indication that the network is successfully learning the classification task. The agreement of accuracies reported during the training and testing data sets indicates that the network is successful in generalizing the problem and not performing memorization of the training patterns (avoiding overfitting). The patterns shown in red describe the error functions for the training and testing data sets (dashed and solid lines, respectively). A decreasing value of the error function is a further indication of successful learning, with a gradually plateauing pattern that indicates a saturated training session. Table 3 summarizes the performance metrics for the final trained neural network that used a fixed window size of 150 units. The accuracy, precision, recall, *F*-measure, and specificity, as described by the equations 1, 2, 3, 4, and 5, respectively, are presented in Table 3. The test accuracies for eating, jogging, medication, and smoking were 94.3%, 100%, 93.6%, and 98.6%, respectively. The average performance attained was 96.6%. To explore the full nature of misclassifications, the confusion table was examined (Table 4).

**Table 3:** Summary metrics for the best performing configuration of window size of 150 units.

Activity	Participants, n	Precision (%)	Recall (%)	*F*-measure (%)	Specificity (%)	Accuracy (%)
Medication taking	28	96.1	92	94.3	95.1	93.6
Eating	6	80.2	92	85.7	94.8	94.3
Smoking	12	88.8	76.9	82.4	99.6	98.6
Jogging	27	100	100	100	100	100
Average	N/A^a^	91.3	90.3	90.6	97.4	96.6

^a^N/A: not applicable.

**Table 4:** Medication-taking protocol (MTP) or non-MTP confusion matrix for the window size of 150 units. The configuration produced the best performance among the different models.

		Predicted label, n (%)	
		ANN^a^ MTP (n=7683)	ANN non-MTP (n=12,932)
**True label**			
	True MTP	7412 (96.5)	708 (5.5)
	True non-MTP	271 (3.5)	12,224 (94.5)

^a^ANN: artificial neural network.

## Discussion

### Principal Findings

This study aimed to examine the feasibility of detecting nMTEs using smartwatches. Studying human activities using smart and wearable devices has numerous advantages over the traditional approaches. Wearable devices provide the advantage of unobtrusively, continuously observing human behavior in their natural settings, with little burden on the user. The collected sensor data from these devices can be used to validate the data reported by the user, thereby improving the accuracy and completeness of the self-reports. In this study, participants used the smartwatch to report the beginning and end of their MTEs. Errors in the participants’ self-reported MTEs were identified. Some self-reported MTEs had implausibly short or long durations. By validating the digitally recorded temporal gestures, we demonstrated the ability to correct erroneous self-reports, thereby improving the quality of the reports. Furthermore, the temporal signature that has been reported by the array of sensors available on wearable devices can provide a plethora of additional information such as the temporal variation of an activity for a given user or across a population of users. For example, in our study, we demonstrated that nMTEs were completed in an average of 18 seconds, but there were distinct differences across participants. Such a comparison of behaviors provides several dimensions along which the study of human behavior can be expanded.

While the ANN’s overall best performance of 96.6% accuracy in identifying MTEs from other activities was good, there are other nuanced configurations that can be explored. In addition to the sliding window size, it is possible to manipulate the hyperparameters, such as the learning rate, number of LSTM units, window step sizes, and batch sizes, to achieve an even better performance. We will consider these in future experiments. In addition to the expanded information that can be obtained from these devices, the ability to automatically detect and identify an nMTE with high accuracy will be beneficial. The automatic detection of an MTE event can be used as the foundation for both monitoring MTEs and improving medication adherence. In the latter case, the nondetection of an nMTE offers the opportunity to alert patients or support persons who missed medications to improve adherence and the health outcomes associated with improved medication adherence. Improved medication adherence has the potential to significantly reduce morbidity [60-62], mortality [3,62], and health care costs [60,63-65]. Hence, detecting nMTEs using smartwatches has exponential utility.

This study addressed several gaps and limitations of other studies. Fozoonmayeh et al [66] used Android LG Watch Sport smartwatches with cellular capability, eliminating the need for a smartphone to transfer data to the cloud repository. The study also relied on both the accelerometer and gyroscope sensor data to detect motions. Data collection and subsequent transmission to the cloud storage were completed in real time, requiring the watch to have constant data connectivity. Constant connectivity uses large amounts of energy; therefore, battery life may be problematic and data integrity can be compromised by poor or noisy connectivity. Our study simplified data collection by adopting an offline approach. However, it relies on a paired smartphone to relay the data to cloud storage. Importantly, our smartwatch app, MedSensor, performs preliminary data annotation at the edge, that is, the data origination point, which makes further downstream automation easier.

The Medhere study [39] addressed poor medication adherence by using a smartwatch accelerometer and gyroscope sensors to monitor a series of actions. While the study also considers the medication activity as a complex activity composed of atomic activities, it applies random forest ML algorithm to classify several discrete actions, including medication intake, with an average precision and recall across all activities of 0.91 and 0.93, respectively. Our study implemented an LSTM network algorithm with a similar average precision and recall of 0.91 across all activities. The LSTM injects the benefit of context as well as the architecture that suits the time series of human activity data. The Medhere study engaged 5 participants; in contrast, MedSensor engaged a larger diverse group of 28 participants.

A separate study used the Kinect depth camera to automatically generate templates for signal matching during the training phase of an inertial sensor [67]. Only 2 actions (“twist-cap” and “hand-to-mouth”) were tracked by the inertial sensor to identify the individual pill-taking signatures among the 5 participants. The approach required a Kinect depth camera during training, and the training was user specific. Despite the generally accurate prediction scores, user-specific training does not lend itself to practical scalability or generalizability across users.

Other notable medication adherence approaches include the following: (1) the AdhereTech platform focuses on a stand-alone cellular enabled bottle that transmits data in real time when it is opened and incorporates SMS text messaging, phone calls, and chime alerts and reminders to patients so that they do not forget to take their medicine; (2) the Vitality GlowCap system works with a smart cap that attaches to a medication bottle and sends alerts to patients when to take their next dose; and (3) PillsyCap is a smart-pill cap for prescription drugs [68]. The PillsyCap uses Bluetooth to synchronize with a mobile app. Although these approaches make it possible to adhere to medication requirements, human activity recognition relies on objects that are not worn on the human body. The reusability of objects, such as smart caps, is another drawback because each bottle must have its own cap. The cost of production of such objects is ultimately higher than that of relying on reusable wearable sensor devices, such as smartwatches. In addition, smartwatches have multiple capabilities that make them appealing as affordable and useful devices.

### Limitations

The limitation of our approach, given the current state of the technology, is the availability of data from a single hand. Therefore, activities such as smoking that can be completed by a single hand, may not be detected by a watch that is worn on the opposite hand. Fortunately, because activities such as medication taking are difficult to complete with a single hand, a residual portion of the activity will always be present from the perspective of a single watch. This problem can be easily overcome with the presence of a sensing device on each hand. Although not common presently, the arrival of smart wristbands, rings, and other forms of wearable devices is likely to provide a more complete picture of a person’s daily activities [27,30,33,35,69].

The second limitation is the method by which people may wear their watches. A watch can be worn in 4 distinct ways on the left or right hand and inside or outside of the wrist. In this study, we asked participants to wear their watch on their right hand and on the outside of their wrists. However, sensor data recorded by the same watch in any of the other 3 configurations will produce related but undistinguishable signals by the ANN. Consistently wearing a watch on the outside right-hand side is critical at the current stage of our scientific development. However, using the existing human body symmetry and the relationship between the inside and outside of the wrists, mechanisms of unifying sensor signals collected from any mode of use can be developed as demonstrated previously [20]. This will produce high-capacity models with broader experience to recognize medication gestures regardless of the watch-face orientation.

### Future Implications

Our current protocol performed well. However, our future investigations will benefit from 2 additional steps to our existing protocol. The first step will collect calibration data during the initial orientation session. Currently, our orientation consists of familiarizing the participant with the watch, app, and use of the app. In the future, we will incorporate a second step, which will use this orientation session to collect data from a set of simple activities (eg, touch toes, touch hips, and touch head) with the watch worn on each hand to obtain useful participant-specific data at baseline. By collecting data from left and right hands, we can establish a more precise relationship between the right and left hands for the given participants. Although perfect human symmetry may indicate a 180° rotation between the 2, natural human posture may create a departure from the ideal 180° symmetry. This information can be used to allow the user to wear the watch in any preferred method and provide the necessary information for the correction that is needed for the existing right-handed ANN. We intend to add this step to our protocol and conduct a future study with participants who are taking their own medications in their natural environment.

In addition, as an ultimate objective, we aim to develop 1 application that can decipher numerous human activities to establish correlative or causative relationships between activities. For instance, medication-taking activity may occur at 8 PM before sleep activity or 7 AM before breakfast eating activity, or eating activity at 1 PM may subsequently be followed by cigarette smoking. The ability to monitor the temporal relationship between these events would be useful to provide the much-needed context to further understand human behavior and, therefore, to model useful health-related solutions or provide real-time intervention reminders. To accomplish this, we need to engage in a formal investigation of the optimal viewable window size for an LSTM that is sufficient to successfully decipher all activities of interest. In addition, there exists some inherent parallelism between human activities and the principles of written language. To fully leverage this parallel analogy, human activities need to be examined in the more fundamental fashion by decomposing complex activities further into their most basic building blocks, referred to in this study as mini-gestures. Our previous work [15,48] illustrates the mini-gesture decomposition of the eating activity in relation to other similar activities such as smoking. Furthermore, our study also compared the medication activity against the jogging activity, and our models confirmed little confusion between medication taking and jogging. This could be explained by the fact that the mini-activities of both complex dynamic activities are largely different. Based on this observation, we speculate the accuracy of the system to increase notably if other natural daily activities are included in our training and testing sets because of their dissimilarity to medication-taking gestures. Thus, our future goal is to develop a powerful and useful application that can distinguish between a multitude of different activities by recognizing the combined mini-gestures or mini-activities of more complex behaviors and temporal relationships.

### Conclusions

Medication adherence is a complex human behavior that is associated with chronic condition self-management. It remains a global public health challenge, as nearly 50% of people fail to adhere to their medication regimens. Automated detection of medication-taking activities is of critical importance for improving treatment effectiveness. The automatic detection of medication-taking gestures will also help eliminate the burden of self-reporting from the participants and provide a simpler method of tracking MTEs. In this study, we demonstrated the use of LSTM to detect and recognize mini-activities and complex activities. We have demonstrated successful identification of individual medication gestures with an accuracy of approximately 93.6% when tested against activities that substantially resemble medication taking, such as smoking and eating, which share the common mini-gestures of hand-to-mouth, hand-on-mouth, and hand-off-mouth. Furthermore, we demonstrated the ability of the neural network to distinguish MTEs from the similar activities of eating and smoking and from the dissimilar activity of jogging. Our future work will build on these successes by making small changes to the protocol and further tuning the network hyperparameter values. In the short term, we anticipate testing MedSensor with people who are taking medications in their natural environment, and our long-term goal is to develop a comprehensive app that can accurately identify a multitude of human behaviors.

## Abbreviations

AI: artificial intelligence
ANN: artificial neural network
CDC: Centers for Disease Control and Prevention
FN: false negative
FP: false positive
LSTM: long short-term memory
ML: machine learning
MTE: medication-taking event
nMTE: natural medication-taking event
RNN: recurrent neural network
sMTE: scripted medication-taking event
TN: true negative
TP: true positive
